# Surface Acoustic Wave-Based Microfluidic Device for Microparticles Manipulation: Effects of Microchannel Elasticity on the Device Performance

**DOI:** 10.3390/mi14091799

**Published:** 2023-09-21

**Authors:** Gianluca Mezzanzanica, Olivier Français, Stefano Mariani

**Affiliations:** 1 Department of Civil and Environmental Engineering, Politecnico di Milano, Piazza Leonardo da Vinci 32, 20133 Milan, Italy; stefano.mariani@polimi.it; 2 Electronics, Communication systems and Microsystems (ESYCOM), Université Gustave Eiffel, National Centre of Scientific Research (CNRS), F-77454 Marne-la-Vallée, France; olivier.francais@esiee.fr

**Keywords:** acoustofluidics, surface acoustic waves, finite element analysis, PDMS elasticity

## Abstract

Size sorting, line focusing, and isolation of microparticles or cells are fundamental ingredients in the improvement of disease diagnostic tools adopted in biology and biomedicine. Microfluidic devices are exploited as a solution to transport and manipulate (bio)particles via a liquid flow. Use of acoustic waves traveling through the fluid provides non-contact solutions to the handling goal, by exploiting the acoustophoretic phenomenon. In this paper, a finite element model of a microfluidic surface acoustic wave-based device for the manipulation of microparticles is reported. Counter-propagating waves are designed to interfere inside a PDMS microchannel and generate a standing surface acoustic wave which is transmitted to the fluid as a standing pressure field. A model of the cross-section of the device is considered to perform a sensitivity analysis of such a standing pressure field to uncertainties related to the geometry of the microchannel, especially in terms of thickness and width of the fluid domain. To also assess the effects caused by possible secondary waves traveling in the microchannel, the PDMS is modeled as an elastic solid material. Remarkable effects and possible issues in microparticle actuation, as related to the size of the microchannel, are discussed by way of exemplary results.

## 1. Introduction

In recent years, the development of simple, effective, and low-cost techniques able to provide precise manipulation of micro and nanoparticles, such as polystyrene microbeads and cells or their aggregates, has gained great attention in the biomedical and biochemical fields [1,2,3,4,5,6]. The great advantage of these solutions is linked to the possibility of manipulating biological species without the use of biomarkers and antigens [7], which can disrupt the cells.

Manipulation techniques have exploited the difference between the physical properties of the particles or cells to be sorted or isolated [8] and the background medium ones. Accordingly, cells can be isolated based on their size, density, deformability, and surface protein expression (in the case of cell functionalization) [9]. Among the different cell separation techniques, the most common ones include immunomagnetic cell separation, fluorescence-activated cell sorting, density gradient techniques, and immuno-density cell isolation [10,11,12,13]. These techniques are all effective but also time-consuming and sometimes complex to perform. 

Recently, microfluidic techniques have been introduced to fulfill the same task. Microfluidics grants several advantages regarding the fabrication of biomedical devices aimed at cell or microparticle handling [14]. These advantages include, besides the possibility to fabricate low-cost and disposable devices, the use of small volumes of fluids and chemical reagents, a simple and precise manipulation of the particles, a good throughput, in situ real-time observations, and the integration on the same device of, e.g., fluidics, manipulation technology, chemical reaction spots, and the particle counting system [15,16,17]. Moreover, the flow regime in channels of micrometric dimensions is usually laminar due to the small value of the resulting Reynolds number, so that control over the flow gets mastered. One recently exploited technology to cover this kind of microfluidic device, termed lab-on-a-chip (LoC), is largely used in biomedical and biochemical research for fast and versatile testing [18,19]. 

Several microfluidic techniques have been reported to be efficient in manipulating the microparticles. They are classified as hydrodynamic, exploiting channel geometry or positioning obstacles; electrical; optical; or magnetic. Relevant examples are reported in [20,21,22,23,24]. Some of these techniques require labeling, a chemical modification, or isotope substitution that makes the identification of a molecule possible; others are label-free and are therefore simpler to perform without markers to detect the particles [25]. Among them, a non-contact, gentle, and biocompatible solution is the acoustic manipulation technique [26]. In particular, the acoustic-based technique is active, due to the use of externally generated ultrasound waves, and label-free, only exploiting for manipulation the properties of the particles such as size, density, and compressibility [27].

The acoustic manipulation method is based on the exploitation of a pressure acoustic field within the fluid contained in a microchannel, which acts on the particles to displace them. Two main phenomena are induced by an acoustic pressure field [28,29]: Acoustophoresis, namely acoustic migration; and acoustic streaming, a steady flow effect generated in a fluid interacting with the acoustic waves. A precise acoustic-based manipulation can be achieved by generating a standing pressure field in the microchannel. The waves are scattered by their interaction with the particles, which, as a response, are subjected to an acoustophoretic radiation force (ARF) [30]. The flow of the fluid through the microchannel induces another force on the particles, the Stokes drag force, which transports the particles in the flow direction from the inlet to the outlet and is proportional to the velocity of the fluid itself [31]. The cooperative effect of these two forces provides the possibility of moving particles or cells inside a microchannel. Moreover, the flow of a fluid in a microchannel causes the development of hydrodynamic forces pushing the particles towards the central streamlines of the flow itself [7].

Bulk acoustic wave (BAW)-based resonators and surface acoustic wave (SAW)-based devices have been reported as possible alternatives to transmit the acoustic waves to a fluid [32,33,34]. In particular, surface acoustic waves can be generated by applying an alternated voltage signal to a series of finger electrodes, called inter-digitated transducers (IDTs), patterned on the surface of a piezoelectric substrate [35]. In this way, the inverse piezoelectric effect is activated to convert the electric input to the IDTs into a mechanical output consisting of surface waves traveling across the substrate [36]. When these surface waves interact with the liquid, its viscosity causes part of the SAWs to refract into the liquid as a longitudinal wave. The mode of SAWs changes to a form called leaky SAW, which moves in the fluid along a direction tilted by the Rayleigh angle $\theta_{R}=arcsin\left(c_{l}/c_{s}\right)$, where $c_{s}$ and $c_{l}$, respectively, denote the velocities of the waves in the piezoelectric substrate and in the liquid medium [34]. 

Two or more counter-propagating waves, generated by different IDTs and traveling in different directions, can constructively interfere to generate a standing surface acoustic wave (SSAW) [37]. The pressure field induced inside the fluid is, in this case, a standing one composed of pressure nodes, characterized by zero wave amplitude, and antinodes, characterized instead by the maximum wave amplitude, with a time-independent pattern. Considering such a standing field, the ARF direction is defined by the physical properties of the particles; typically, solid particles are displaced towards the pressure nodes of the standing pressure field [38].

Microfluidic acoustic devices can be utilized for cell/particle sorting, isolation, patterning, focusing, and enrichment [39,40,41,42,43]. Applications towards biological cell focusing (for example, cell counting) and size-based separation of circulating tumor cells from other major blood cells for disease diagnoses, can be achieved using this manipulation method.

In this work [44], a two-dimensional (2D) numerical analysis of a microfluidic SSAW-based device is performed to study the acoustofluidic phenomenon, hence the coupling between acoustics and fluidics and the exploitation of the pressure acoustic field to manipulate microparticles. The cross-section of the device is modeled, and the interaction between mechanics, acoustics, and fluidics is assessed in the frequency and time domains. The behavior of the materials involved, polydimethylsiloxane (PDMS) and lithium niobate (LiNbO_3_), is investigated to understand the sensitivity of the pressure field to geometrical imperfections, like those that are accidentally influenced by the fabrication of the microchannels. A parametric study is performed for this purpose by varying the thickness and width of the microchannel. The PDMS is modeled as a solid elastic material to also gain insights into the transmission of secondary waves to the fluid, besides those transferred from the substrate. To tackle the soft material nature of the PDMS, the effect of wave damping on the acoustic field distribution is considered. Ni et al. [45] already reported different approaches for PDMS modeling in acoustofluidic devices; their analysis highlighted that the modeling of PDMS as an elastic (solid) continuum, supporting the propagation of shear waves, causes specific acoustic streaming effects at the fluid/PDMS interface boundary layer, so that the acoustofluidic problem can be approached more accurately. Considering particles below a certain dimension (typically submicron sized [46]), the acoustic streaming force has a greater effect on particle manipulation with respect to ARF. In our analyses, due to the considered micrometric size of polystyrene (PS) spherical microbeads, results in terms of their focusing towards the pressure nodes by the acoustic radiation are reported, while the effect of the acoustic streaming is neglected. Specifically, some exemplary cases taken out of the aforementioned sensitivity analysis are shown to highlight the importance of properly modeling the PDMS as a solid material to bind the acoustofluidic microchannel and fully provide a solution in terms of the acoustic pressure distribution.

## 2. Numerical Model

A 2D numerical model of the cross-section of the complete device is considered. 2D models of SSAW devices have been widely reported to study the acoustophoretic phenomenon [37], to investigate the microchannel behavior [45], and to compare the performance of hard wall channels with respect to soft wall ones [46]. Here, a study on the sensitivity of the standing pressure field to variations in the thickness and width of a soft PDMS microchannel cavity is performed as a preliminary step before the fabrication of the device. The addressed task is to understand the elastic response of the PDMS microchannel to the SSAW and how it can affect the acoustic radiation pressure field within the fluid, which is strictly correlated with the manipulation of the microparticles. This study will contribute to the optimization of the acoustofluidic device geometry and the improvement of its performance.

The finite element analyses have been performed using COMSOL Multiphysics^®^ v6.0 (Stockholm, Sweden) [47], which allows to properly account for the coupled multiphysics governing the working conditions of the device.

**Figure 1:**
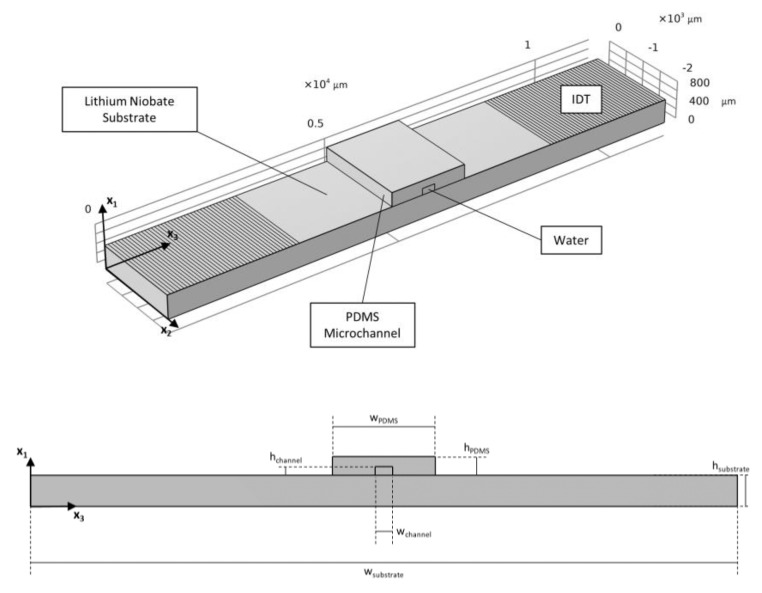
Central portion of the SAW-based system, and 2D cross-section exploited to perform the analyses.

A part of the considered SAW-based system is depicted in Figure 1. Its cross-section has been exploited for modeling the phenomenon. The substrate is a plate made of LiNbO_3_, 500 *μ*m thick. Another widely used piezoelectric material for the generation of SAWs is lithium tantalate (LiTaO3), which exhibits almost the same properties as lithium niobate but with a slightly worse coupling between the mechanical and electric fields [48]. For this reason, lithium niobate has been chosen. On its surface, two IDTs are patterned on the opposite sides of the channel. The finger electrodes composing the IDTs are assumed to be made of gold, 70 *μ*m wide, and with a spacing between them of 70 *μ*m. The fingers are parallel to the lateral surfaces of the channel. Both IDTs are composed of 10 pairs of electrodes to ensure proper actuation of the substrate and the formation of the SAWs. The IDTs generate two traveling SAWs (TSAWs), which constructively interfere developing an SSAW within the channel. The microfluidic straight channel has the goal of transmitting the standing waves to the fluid flowing inside it. The channel is obtained by molding PDMS, a largely used soft polymeric material due to its optical transparency, ease of handling, and biocompatibility [49]. The chosen design for the microchannel allows for the display of one pressure node of the standing wave in correspondence with its longitudinal axis. Being the SAW wavelength *λ*_SAW_ equal to the pitch of the IDTs, namely the distance covered by a pair of electrodes, its value results in being 280 *μ*m. To generate an SSAW featuring a node at the central point of the microchannel, the two counter-propagating TSAWs should interact after covering a distance of *nλ*_SAW_ with, in this case, *n* = 10. Regarding the domain in the microchannel, the fluid is assumed to be water, and the released spherical particles are made of PS. 

In COMSOL Multiphysics^®^, to model the SAW-based microfluidic device, the following modules have been exploited: piezoelectricity, to couple electrostatics and solid mechanics; pressure acoustics; and particle tracing. Besides them, in a truly three-dimensional (3D) model, the (laminar) fluid flow from the microchannel inlet should also be considered.

A linear piezoelectric constitutive model has been assumed to govern the mechanical response of the lithium niobate when an electric stimulus (voltage difference between successive IDT electrodes) is applied. As the SAWs represent a (small) perturbation to the state of the substrate, the equations describing the piezoelectric effect read, in a stress-charge form:

(1) $$T=C^{E}\cdot S-e^{T}\cdot E$$

and

(2) $$D=e\cdot S+\varepsilon^{S}\cdot E,$$

where a Voigt notation has been adopted, see [50]. The considered orthonormal reference frame is depicted in Figure 1 and: $T={\left\{T_{11},\;T_{33},\;T_{13}\right\}}^{T}$ is the stress vector; $C^{E}$ is the elasticity matrix, at constant electric field E; $S={\left\{S_{11},\;S_{33},\;{2S}_{13}\right\}}^{T}$ is the strain vector; e is the piezoelectric coupling matrix; D is the electric displacement vector; and $\varepsilon^{S}$ is the electric permittivity matrix, defined at constant strain S; the superscript T means transpose. Due to the lithium niobate being anisotropic, the elasticity matrix $C^{E}$ and the coupling matrix e are populated with different coefficients, depending on the orientation. The model has been solved to account for the so-called 13-effect, so that:

(3) $$C^{E}=\left[\;\begin{array}{ccc} c_{11} & c_{13} & 0\\ c_{13} & c_{33} & 0\\ 0 & 0 & c_{55} \end{array}\right],$$

(4) $$e=\left[\begin{array}{ccc} 0 & 0 & e_{15}\\ e_{31} & e_{33} & 0 \end{array}\right].$$

The electric permittivity matrix $\varepsilon^{S}$ is instead diagonal, namely:

(5) $$\varepsilon^{S}=\left[\begin{array}{cc} \varepsilon_{11} & 0\\ 0 & \varepsilon_{33} \end{array}\right].$$

The out-of-plane $T_{22}$ component of the stress vector T is computed to satisfy the plane strain condition $S_{22}=0$. 

As already pointed out, by exploiting the inverse piezoelectric effect, surface acoustic waves are generated where the IDTs are patterned. The waves can then propagate without any constraint inside the substrate; to keep their fronts straight and without interferences with the lateral and bottom surfaces of the substrate itself, low-reflecting boundary conditions are considered to lead to a perfect impedance match for both pressure and shear waves. This condition can be reproduced in a real device by attaching an absorbing material to the boundaries or by patterning IDT-like structures designed to avoid wave-back reflection. A stress-free boundary condition is applied to the top surface of the piezoelectric substrate.

The pressure acoustics module has been adopted to compute the pressure variation in the fluid induced by the propagation of the acoustic waves at any frequency in the case of harmonic excitation. The Helmholtz wave equation, to be solved in the frequency domain for given frequencies, reads:

(6) $$\frac{1}{\rho_{i}^{*}}\nabla^{2}p-\frac{\omega^{2}p}{\rho_{i}c_{i}^{2}}=0,$$

where p is the acoustic pressure and $\rho_{i}^{*}$ is defined by:

(7) $$\rho_{i}^{*}=\frac{\rho_{i}c_{i}^{2}}{\omega^{2}}{\left(\frac{\omega }{c_{i}}-j\ln\left(10\right)\frac{\alpha_{i}}{20}\right)}^{2}.$$

In the equations above, $\rho_{i}$ is the density of the fluid (in this case, water); $c_{i}$ is the speed of sound in the fluid; $\omega =2\pi f_{SAW}$ is the angular frequency, while $f_{SAW}$ is the SAW frequency; ∇ is the gradient operator; and $\alpha_{i}$ is the acoustic attenuation coefficient associated with the fluid [51]. The acoustic pressure in the fluid induced by the SAWs is obtained by coupling the fluid load on the substrate with the acceleration experienced by the fluid. This coupling is defined as a continuity between the mechanical stress T and acoustic pressure p, according to:

(8) T·n=−p·n,

(9) $$n\cdot \left(-\frac{\nabla p}{\rho_{i}^{*}}\right)=a_{n},$$

where n is the surface normal vector; $a_{n}$ is the structural acceleration along the n direction; and ∇p is the pressure gradient. The same condition is applied to the surfaces that couple the solid elastic PDMS and the fluid, to transmit the waves from the microchannel structure to the fluid. A thorough description of the coupling between solid and fluid fields is reported in [51,52].

To account for the viscoelastic nature of the PDMS, which attenuates the propagation of elastic waves, an isotropic loss factor $\eta_{s}$ has been added. This factor is set to modify the elastic stiffness matrix according to [53]:

(10) $$C^{E}\to C^{E}(1+i\eta_{s}),$$

i being the imaginary unit. 

The same PDMS boundary conditions applied by Ni et al. in [45] have been adopted in the presented model. The top lids of microchannels are typically several millimeters in thickness; therefore, the reflected waves from the top surface of the microchannel lid can be neglected due to wave attenuation. To model that effect, the PDMS upper surface is assigned to be low-reflective. In this way, the thickness of the lid can be reduced in the model, and the computational performance is improved. Both the lateral surfaces of the microchannel structure are set as traction free.

In order to highlight the effect of the elastic waves in the PDMS on the fluid acoustic pressure field, the solid material model is compared with another model description reported in the literature. The acoustic pressure transmission from the soft material is also achieved by modeling the PDMS as an absorptive non-flowing fluid, see [45], to also account for its viscous nature. Even if not investigated in this work, the PDMS could also be modeled by setting an acoustic impedance at the fluid domain interface.

Like the computation of the acoustic pressure in the water domain, the Helmholtz equation, see Equation (6), is exploited to provide it in the non-flowing fluid-modeled PDMS (model A in reference [45]). At the boundaries, an impedance boundary condition is applied in the form:

(11) $$n\cdot \nabla p=-i\frac{\omega \rho_{i}}{\rho_{m}c_{m}}p,$$

where $\rho_{m}$ and $c_{m}$ are the density and the wave speed of the modeled interface material. In this case, the lateral boundaries of the PDMS domain are associated with an impedance boundary condition where the mimicked material is air. The upper boundary is instead set to simulate the presence of a thicker PDMS structure. All the boundaries of the water domain must be associated with the impedance condition, mimicking the presence of the PDMS, in case the microchannel structure is not modeled. In Section 3.5, a comparison between the two solutions (solid elastic and non-flowing fluid media) is reported, discussing the differences in the results in terms of the manipulation of PS microparticles.

For particle tracing, the motion of the particles dispersed in the fluid is governed by Newton’s second law. The acoustophoretic radiation force on small particles, as due to acoustic radiation, is computed as:

(12) $$F^{AR}=-\frac{4\pi }{3}r^{3}\nabla \left[\frac{1}{2}\left(f_{1}\right)\kappa_{i}\langle p^{2}\rangle -\frac{3}{4}(f_{2})\rho_{i}\langle v^{2}\rangle \right],$$

where r is the radius of the particles; $\kappa_{i}$ is the fluid compressibility; $\langle p^{2}\rangle$ and $\langle v^{2}\rangle$ are the time average, over a full oscillation, of the acoustic pressure and velocity fields, as generated by the acoustic waves. In Equation (12), $f_{1}$ and $f_{2}$ are the in-phase components of the monopole and dipole scattering coefficients, which depend on the density and compressibility ratios between particles and fluid according to:

(13) $$f_{1}\left(\tilde{\kappa }\right)=1-\tilde{\kappa }$$

and

(14) $$f_{2}\left(\tilde{\rho }\right)=\frac{2(\tilde{\rho }-1)}{2\tilde{\rho }+1},$$

where $\tilde{\rho }=\rho_{p}/\rho_{i}$ and $\tilde{\kappa }=\kappa_{p}/\kappa_{i}$; the subscript “p” denotes the material properties related to the particles.

The microparticles are respectively attracted towards acoustic pressure nodes or antinodes based on the positive or negative sign of the acoustic contrast factor ϕ, which depends on the $f_{1}$ and $f_{2}$ coefficients and reads:

(15) $$\phi \left(\tilde{\rho },\tilde{\kappa }\right)=\frac{1}{3}f_{1}\left(\tilde{\kappa }\right)+\frac{1}{2}f_{2}\left(\tilde{\rho }\right)=\frac{1}{3}\left[\frac{5\tilde{\rho }-2}{2\tilde{\rho }+1}-\tilde{\kappa }\right].$$

A complete description of the scattering theory related to the acoustic radiation force acting on small particles has been reported by, e.g., Bruus [30].

The presented 2D model lacks a description of the fluid flowing through the microchannel. In real devices, the fluid flow causes the transport of the microparticles from the inlet to the outlet of the microfluidic system. Another force is therefore acting on the particles, the Stokes drag force, which reads:

(16) $$F_{d}=-6\pi \eta rv.$$

in which η is the dynamic viscosity of the fluid and v is the flow velocity relative to the particle. Moreover, the flow generates a hydrodynamic force that tends to push the microparticles in the central region of the channel cross-section while moving. It is important to highlight this effect in cases where the main task of the microfluidic system is the focusing of particles in a single line.

## 3. Results and Discussion

The purpose of this work is to highlight the importance of modeling soft materials used for the fabrication of microfluidic channels, as solid and dissipating ones, to fully assess the acoustofluidic phenomenon in terms of wave transfer to the fluid.

To investigate the generation, propagation, and transmission of the SAWs to the fluid, a time-dependent analysis was first performed. Then, a parametric analysis in the frequency domain has been performed by varying the thickness and width of the microchannel, namely the vertical and horizontal dimensions of the fluid domain, to estimate the sensitivity of the acoustic pressure field to geometrical imperfections that could arise in the fabrication process. The effect of the PDMS response on the wave pattern transmitted to the fluid has been finally inspected to understand the possible interaction between pressure waves and microparticles.

The values of the material properties adopted in the analyses are reported in Table 1. The actuation frequency of the device has been computed, accounting for the speed of the Rayleigh waves $c_{R}$, as: $f_{SAW}=c_{R}/\lambda_{SAW}$ = 12.518 MHz. The value of the isotropic damping factor associated with the PDMS has been chosen as the same adopted by Skov and Bruus in [54]. Finally, the voltage amplitude applied to the IDT electrodes has been set to 8 V, as reported in, e.g., [51].

The geometry of the device has been designed in order to guarantee the presence of an SSAW featuring a node in the central coordinate of the cross-section. The width of one electrode and the spacing between two successive electrodes have a value of $w_{e}$ = 70 *μ*m. Therefore, the pitch of the IDT, corresponding to the SAW wavelength, is $s_{IDT}=\lambda_{SAW}$ = 280 *μ*m. The geometry of the PDMS is the same as the one considered by Guo et al. in [51], with $w_{PDMS}$ = 1650 *μ*m, side-to-side width; $h_{PDMS}$ = 300 *μ*m. The thickness of the PDMS domain is limited with respect to the usual dimensions of fabricated microchannels (several millimeters), for the reason reported in the discussion of Section 2.

### 3.1. Generation, Propagation and Transmission of SAWs

The frequency $f_{SAW}$ of the AC signal to be applied to the IDT electrodes has to induce the Rayleigh waves in the lithium niobate. The theory of Rayleigh waves propagation was thoroughly discussed in [55,56] and is not further reported here.

**Figure 2:**
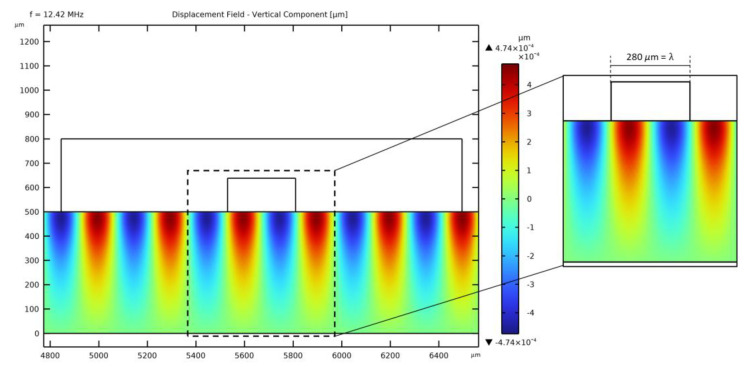
Standing SAW in the region of the PDMS microchannel.

As lithium niobate is an anisotropic material, the specific velocity of waves traveling in the direction perpendicular to the longitudinal axis of the channel has been considered. A frequency domain analysis has been performed by varying $f_{SAW}$ between 12 MHz and 13 MHz to account for the effects of anisotropy on $c_{R}$ and find the correct actuation frequency for the model geometry. The selected frequency, as obtained from the analysis, is $f_{SAW}$ = 12.42 MHz, as also induced by the constructive interference between the opposite TSAWs. In Figure 2, the SSAW in the region of the microchannel is plotted. The wavelength *λ*_SAW_ is assured to be around 280 *μ*m as it fits the width of the fluid domain. The wavelength of the SSAW is the same as that of the traveling waves since they are both actuated at the same frequency $f_{SAW}$, and the sources are placed an integer multiple of *λ*_SAW_ away from each other.

**Figure 3:**
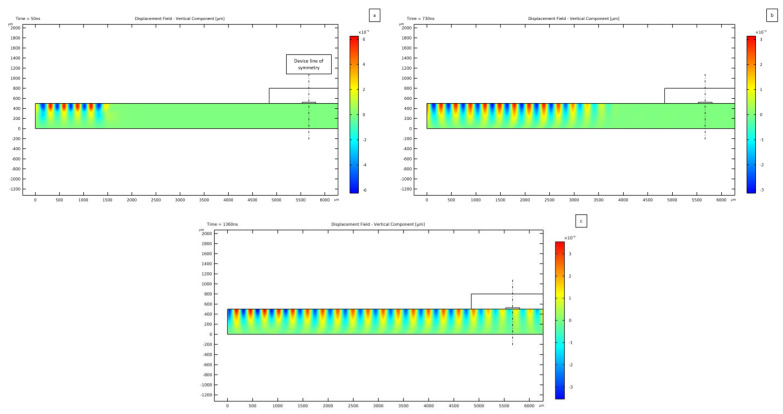
Traveling SAW generation and propagation from one lateral side of the device at (**a**) 50 ns, (**b**) 730 ns and (**c**) 1360 ns after the beginning of actuation. The black dashed line represents the symmetry plane of the device.

Having defined the actuation frequency, time domain analyses have been run with the voltage applied to the electrodes harmonically varying in time between −8 V and 8 V. The generation through the inverse piezoelectric effect and the following propagation of the TSAW at the lithium niobate surface at three different instants after the beginning of actuation are shown in Figure 3. As soon as the IDTs are actuated, the surface waves are triggered below the electrodes (Figure 3a) and then travel across the device cross-section (Figure 3b) to finally meet with the other counter-propagating TSAW (Figure 3c), leading to the reported SSAW solution around the channel. The plots show that the Rayleigh wave is characterized by the largest (vertical) displacements at the top free surface of the substrate; moving downwards, the wave is reduced in amplitude till vanishing at a distance d ≅ 1.5 $\lambda_{SAW}$ = 320 *μ*m away from the surface, where only 10% of the maximum wave amplitude survives. As far as the design of the entire device is concerned, the adopted thickness of the piezoelectric substrate needs to be larger than this distance to ensure that possible negative interference effects are not generated by the boundary conditions at the bottom surface of the substrate itself. To completely avoid the interference of reflected waves from the bottom surface of the substrate, low-reflecting boundary conditions have been applied.

**Figure 4:**
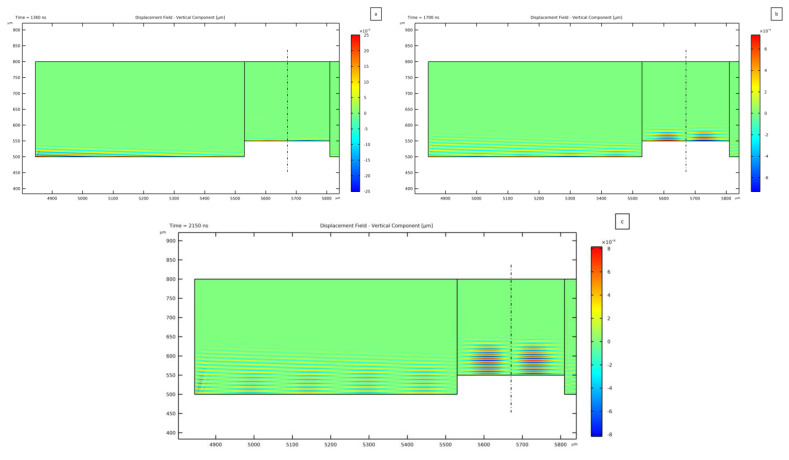
Propagation of the SSAW transmitted from the LiNbO_3_ to the PDMS microchannel. The vertical component of the displacement field is plotted for three successive time steps: (**a**) 1360 ns, (**b**) 1700 ns, and (**c**) 2150 ns from the application of the voltage input to the IDT electrodes.

This time-dependent analysis has been exploited to also assess the transmission of the TSAWs to the PDMS microchannel and to the fluid. In Figure 4, the solution relevant to three successive time steps is reported to show the wave propagation in the PDMS. Initially, the TSAW reaches the PDMS and starts transmitting vibrations inside it. This step is reported in Figure 4a, where the direction of the wave tilted by the Rayleigh angle can be seen. Then, when the two counter-propagating TSAWs start interacting to develop the SSAW, the distribution of the lithium niobate surface wave is reflected in the PDMS; Figure 4b,c report this resulting solution. As can be seen, the region of PDMS above the microchannel shows a vertical propagation of the waves, featuring a zero displacement location at the symmetry line of the device. This effect also results in the distribution of the pressure wave generated inside the fluid, where a pressure node is formed. In the lateral regions of the PDMS, the transmitted wave is instead tilted with respect to the vertical direction. This effect is caused by the propagation of the TSAWs, which move vertically on the substrate surface while propagating in the horizontal direction: this is the reason why the standing acoustic field requires some time to be generated and completely developed.

**Figure 5:**
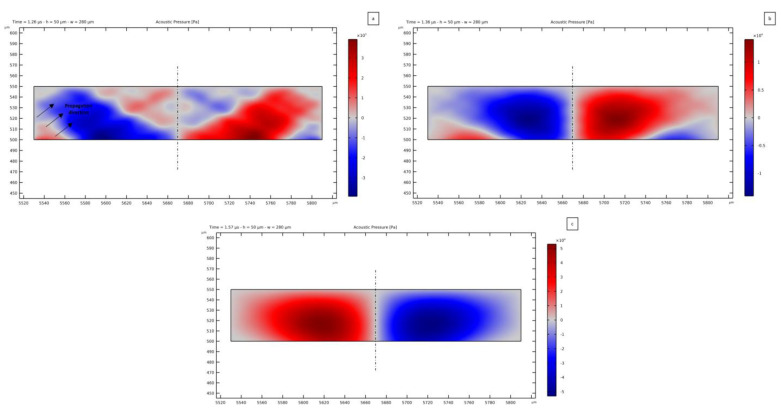
Highlight on the leaky-SAW phenomenon: transmission of the TSAW to the fluid (**a**) 1.26 *µ*s, (**b**) 1.36 *µ*s and (**c**) 1.57 *µ*s after the application of the voltage. The direction of propagation of the pressure waves results to be tilted by the Rayleigh angle.

As a result of the analysis, the transmission of the TSAWs to the fluid in the microchannel is computed. In Figure 5, the leaky SAW phenomenon is shown for three successive time steps of the standing acoustic wave generation. The Rayleigh angle of propagation of the pressure waves inside the fluid can be seen, highlighted by arrows in Figure 5a. The magnitude of the pressure field is initially 0.18 Pa at the activation of the voltage signal input, which is very low with respect to the expected value on the order of 10^4^ Pa for a standing pressure wave. This value increases due to the transmission of an SSAW generated by the constructive interference of the two counter-propagating waves (Figure 5b), and the pressure field in the fluid domain then reaches the expected amplitude after 1.57 *μ*s (Figure 5c) the beginning of actuation. The pressure amplitude can obviously be increased by increasing the value of the voltage applied to the IDTs. From Figure 5 it is clear that the acoustic pressure wave is generated in the fluid as a combination of the vertical motion due to the SAWs and their horizontal propagation. Therefore, the transmission of the waves results tilted with respect to the vertical direction, becoming vertical with the development of the SSAW at the end of the interaction between the two opposite TSAWs (see Figure 5c).

### 3.2. Pressure Field Sensitivity Analysis: Impact of Channel Thickness

Through the formerly described time-dependent analysis, the potential effect of the acoustofluidic interaction has been assessed.

Then, a parametric analysis has been run in the frequency domain by varying the thickness and width of the fluid domain. Specifically, the thickness $h_{channel}$ has been varied between 5 *μ*m and 150 *μ*m with a step of 1 *μ*m, while the width $w_{channel}$ has been varied between 140 *μ*m and 420 *μ*m with a step of 1 *μ*m. The thickness and width of the PDMS domain have instead been kept fixed to the values presented at the beginning of this section.

The goal of this investigation is to establish anomalies in the development of the standing pressure acoustic field caused by the interaction between the waves in the solid elastic PDMS and the fluid domain contained in the microchannel. This undesired effect can be studied, detected, and avoided in device fabrication using PDMS as a molding material.

Moving from the work in [45], the PDMS has been modeled as a linear elastic solid material. This material behavior seems to provide more accurate results if compared to non-flowing fluid modeling; in fact, in this latter case, the continuity of the pressure wave between the fluid and PDMS is imposed to allow wave transmission. In a real device, the vibration of the piezoelectric substrate is transmitted to the material of the microchannel structure, which also constrains the waves along its two lateral surfaces. The aforementioned anomalies could not be reported by modeling the PDMS as a fluid due to the missing acoustic-mechanics coupling between the fluid and the PDMS.

The figures in this section and the following ones report the acoustic pressure field within the fluid domain contained in the microchannel. The reference frame is different in each figure in order to fit the whole domain into the frame itself and highlight details of the solutions. Regarding the geometry of the channel, in this sub-section, the only dimension that is modified is the thickness of the fluid domain, keeping the width fixed to $\lambda_{SAW}$. In the next sub-section, different widths are considered for only two values of thickness: 50 *μ*m and 138 *μ*m.

**Figure 6:**
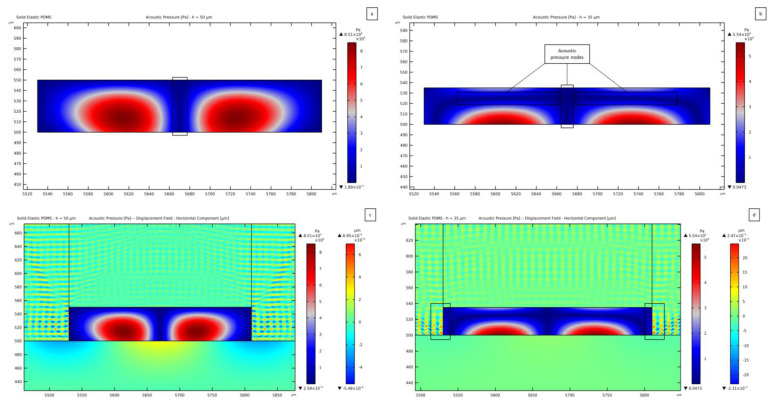
Standing acoustic pressure fields relevant to: (**a**) 50 *µ*m thick domain, featuring a regular pressure distribution; (**b**) 35 *µ*m thick domain, with secondary waves in the PDMS causing an anomalous distribution of nodes and antinodes. Plot of the horizontal component of the PDMS displacement together with the resulting standing pressure field: (**c**) 50 *µ*m thick domain; (**d**) 35 *µ*m thick domain.

In Figure 6, the standing pressure fields related to different thicknesses of the microchannel (namely 35 *μ*m and 50 *μ*m) are reported.

The goal being a one-to-one link between the SSAW and the pressure wave in the fluid domain, the width of the channel must be equal to the SAW wavelength $\lambda_{SAW}$ to display two antinodes at the lateral sides of the fluid domain, placed symmetrically alongside one single node at mid-width. In Figure 6 and those to follow, the red regions of the standing pressure wave plots represent the antinodes, while the dark blue regions correspond to the pressure nodes, which are also highlighted by black boxes. These spots are expected to be the attractors of PS microparticles, as caused by the acoustic pressure. In Figure 6a, showing the results relevant to the 50 *μ*m thick channel, the driving force will push the PS particles towards the field nodes; therefore, the pressure distribution is expected to move the particles from the two lateral sides of the channel towards the central region. An anomalous solution can be instead recognized in Figure 6b, which is associated with a PDMS vibration at the top and lateral channel walls that leads to contributions to the pressure waves coming from all the boundaries, see Figure 6d. This solution is characterized by the central pressure node transmitted from the underlying SSAW and also by lateral antinodes, even though a horizontal pressure node shows up on the upper side of the water domain, at variance with the expected solution. This pressure distribution can cause an undesirable motion of the particles. The same anomalous effect is obtained for a thickness value of 36 *μ*m; for smaller and larger $h_{channel}$ thickness values, the distribution is in accordance with the one provided by the SSAW, as reported in Figure 5a. Looking at the maximum and minimum values of pressure, the magnitude turns out to be smaller for the 35 *μ*m thick case with a maximum of 5.54 × 10^4^ Pa, which compares to a pressure magnitude of 8.51 × 10^4^ Pa in the 50 *μ*m thick case. To stress out how the standing pressure wave distribution is developed within the fluid, a comparison between the horizontal component of the displacement in the PDMS (colored in orange and blue, around the standing pressure wave contained in the rectangular channel) can be considered. The pattern of the PDMS waves in Figure 6d at the interface between PDMS and water is more regular than the one in Figure 6c. In the former, a clear horizontal wave (forward-backward motion) can be seen, while in the latter, a clear wave form cannot be distinguished. The PDMS wave reported in Figure 6d pushes the fluid laterally, causing interference with the contribution coming from the substrate SSAW. As far as the magnitude of the induced displacement field is concerned, the maximum value at the substrate surface (which is directly linked to the SSAW) is around 4.74 × 10^−4^ *μ*m, while it amounts to 2.47 × 10^−3^ *μ*m as induced by the horizontally moving waves inside the PDMS, for a channel 35 *μ*m thick. The vibrations in the PDMS are thus 5.2 times greater than the SSAW-induced motion in the lithium niobate. The transmission of one pressure wave from the lateral walls of the channel thereby distorts the pressure field.

This first outcome, showing two different standing pressure wave distributions, confirms that secondary pressure waves can be generated from the channel walls and lid. With the term “secondary waves”, reference is made to the waves that are transmitted from the substrate to the PDMS structure to finally interact with the fluid to generate pressure waves. The term secondary is here used to highlight that they propagate from PDMS instead of being the principal SAWs provided by the piezoelectric substrate. In order to observe them, the material covering the microchannel must be modeled as a solid one, allowing the propagation of shear waves within the material, directly interacting with the fluid.

**Figure 7:**
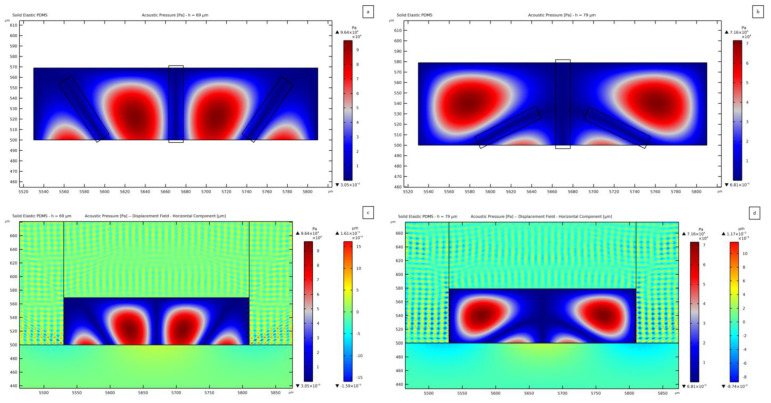
Standing pressure waves anomalies caused by the transmission of the successive half wavelength of the pressure wave in the fluid, relevant to: (**a**) 69 *µ*m, with two more nodes than expected along the width of the fluid domain; (**b**) 79 *µ*m, with the second half of the wavelength being transmitted. Horizontal component of the PDMS displacement shown together with the resulting standing pressure field: (**c**) 69 *µ*m thick domain; (**d**) 79 *µ*m thick domain.

Two other pressure field anomalies are reported in Figure 7. In these cases, the channel thickness results in being critical as its value is close to $\lambda_{p}/2$, being $\lambda_{p}$ the pressure wavelength in the fluid; hence, another wave antinode shows up in the fluid domain through its thickness. The plot reported in Figure 7a refers to a microchannel thickness of 69 *μ*m, but similar distributions develop for thickness values in the range between 60 *μ*m and 73 *μ*m. A distinctive feature of this field is represented by the presence of two additional pressure nodes located near the lateral walls of the channel. This effect is caused by the waves impinging upon the fluid and coming from the lateral walls of the PDMS channel, as reported in Figure 7c, featuring an amplitude 3.4 times greater than the SSAW amplitude. The presence of a second antinode for the pressure field, coming from the substrate motion, is more evident in the solution reported in Figure 7b, where the additional nodes and antinodes emerge directly from the central region of the channel for a 79 *μ*m thick microchannel. The same type of solution is reported for values of the thickness ranging between 74 *μ*m and 88 *μ*m; larger $h_{channel}$ values provide again the target pressure distribution, like for the 50 *μ*m case (see Figure 6a,c). When such a transmission is completely stabilized, a horizontal node can be detected between the two antinodes placed near the top and bottom walls of the channel. On the contrary, with the undesired nodes commented for the previous geometries in Figure 7, by increasing the thickness over 88 *μ*m, the expected horizontal node in the pressure field is obtained.

**Figure 8:**
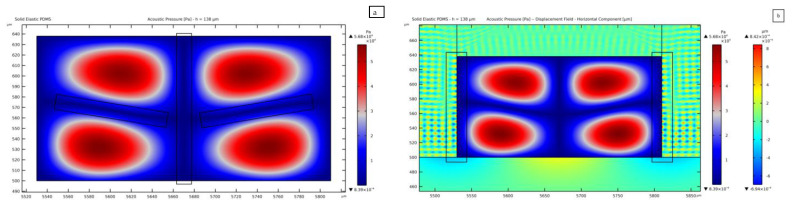
(**a**) One wavelength of the standing pressure wave contained in the fluid domain for the thickness of the channel being 138 *µ*m. (**b**) Horizontal component of the displacement field and standing pressure wave for 138 *µ*m thick microchannel. The black boxes are used to highlight the effect of the PDMS waves on the pressure field distribution.

Figure 8 shows the result related to a 138 *μ*m-thick channel. This plot outcome suggests the idea of designing an innovative configuration of SAW devices where two or more SAWs interact so that the pressure field can allow manipulating particles with control of the pressure distribution in the vertical direction. Not only the focusing of the particles to move from the lateral antinodes, but also the manipulation in the direction perpendicular to the propagation of the wave fronts in the piezoelectric substrate could be then achieved. Regarding the amplitude of vibrations in the PDMS, the horizontal component amounts to 8.42 × 10^−4^ *μ*m, which is twice the SSAW-driven vertical one in the lithium niobate. This response testifies that a certain threshold, in terms of PDMS displacement magnitude, is necessary to overcome the SSAW contribution for transmitting the pressure wave to the fluid domain. This outcome strengthens the classification of these waves as secondary ones.

The target pressure distribution is obtained if the channel thickness varies between 89 *μ*m and 105 *μ*m, with a symmetric distribution, characterized by one vertical and one horizontal node.

**Figure 9:**
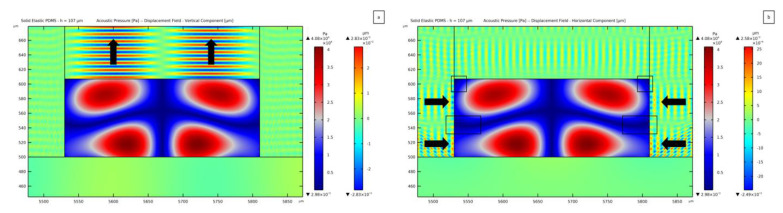
(**a**) Vertical and (**b**) horizontal components of the displacement field within the PDMS, plotted together with the standing pressure field in case of a 107 *µ*m thick fluid domain. The black arrows are used to highlight the direction of propagation of the waves within the PDMS microchannel structure, while the black boxes are used to highlight the effect of the PDMS waves on the pressure field distribution.

The most severe anomaly in the present investigation is obtained for a channel thickness of 107 *μ*m, see Figure 9. To explain such a distortion of the pressure field, the vertical (Figure 9a) and horizontal (Figure 9b) components of the displacement field in the PDMS structure are reported. Looking at these plots, longitudinal waves inside the solid PDMS can be detected. Figure 9a shows that the vertical component of these waves moves in the vertical direction, from the substrate surface towards the top lid of the microchannel; the black arrows in the figure provide an indication of the direction of motion of the waves in the PDMS to transmit the pressure wave within the fluid. This result is totally predictable. The principle of SAW-based devices is to exploit the propagation of surface waves to induce a specific response in the system. A similar behavior can be seen in Figure 9b, regarding the horizontal component of the displacement field. Such secondary waves (as originated from the PDMS and not from the substrate surface) lead to the transmission of pressure waves to the fluid from sources different from the SSAW. The synergic presence of these two motions in the PDMS generates a standing pressure wave field unexpectedly distorted and with a reduced magnitude of 4.08 × 10^4^ Pa, as compared to the field reported in Figure 6a, which features a magnitude of 8.51 × 10^4^ Pa. In Figure 9b, a black box further highlights an interference between the horizontally moving waves in the PDMS, which induces one wavy pressure node in the fluid domain. Due to the limited thickness of the channel, the same effect is partially highlighted close to the top right corner of the channel. The amplitude of the horizontal component of the displacement in this case amounts to 2.58 × 10^−3^ *μ*m; therefore, the vibrations in the PDMS are 5.6 times greater than the motion in the lithium niobate. Hence, as stated before, the distortion of the pressure field can be associated with the lateral transmission of pressure waves.

A different PDMS wave excitation can be distinguished in Figure 8b at the lateral wall of the microchannel if compared to the solution displayed in Figure 9b. While a forward–backward horizontal motion of the PDMS structure can be recognized in the latter (as for the case reported in Figure 6d), the shape of the wave near the lateral wall of the channel is slightly different in the former case (as for the case reported in Figure 6c). The pattern of the PDMS waves in Figure 9b is more regular than the one in Figure 8b. This effect is obtained by accounting for the wave damping in the PDMS microchannel structure, as linked to the parameter $\eta_{s}$. By changing the thickness of the microchannel, the interaction between the horizontally moving waves inside the PDMS changes, causing a distortion of the pressure field.

By increasing the thickness of the PDMS channel even further, a less distorted pattern is obtained until a transition for values larger than 110 *μ*m to a regular distribution, symmetric about the central pressure node.

This first sensitivity analysis has proven the importance of side effects in the design of chip-integrated microfluidic systems. The vibrations of the PDMS channel triggered by the piezoelectric substrate can cause the development of unexpected distributions of acoustic pressure within the fluid. To reduce the presence of pressure field anomalies, the vibrations in the PDMS must be reduced so that they do not exceed a critical magnitude. Critical ranges of the wall thickness have been reported and should be avoided to end up with the requested capability to manipulate the microparticles.

### 3.3. Pressure Field Sensitivity Analysis: Impact of Channel Width

Another parametric analysis is reported here in terms of the results of frequency domain analyses at varying widths of the microchannel. On the basis of the previous results, two values of thickness leading to a regular pressure pattern have been exploited in the parametric analysis: 50 *μ*m and 138 *μ*m. Also in this case, the geometry of the PDMS domain has been kept fixed, as reported at the beginning of Section 3.

**Figure 10:**
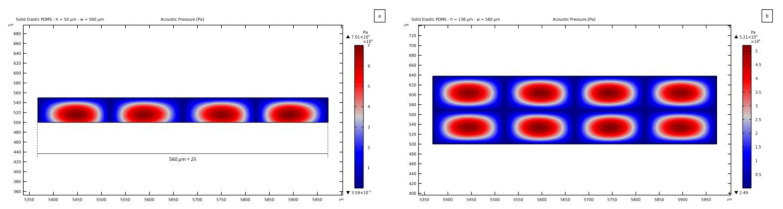
Standing pressure wave distributions for channels featuring a width of 2$\lambda_{SAW}$: (**a**) 50 *µ*m thick channel; (**b**) 138 *µ*m thick channel.

The 50 *μ*m thick case results to be the best as, from the solution of the parametric analysis, the pressure pattern does not depend on the channel width and therefore matches the SSAW-driven one. Figure 10a shows the results in terms of the standing pressure field transmission for a 560 *μ*m wide channel, equal to 2$\lambda_{SAW}$: the maximum pressure magnitude is comparable with the value reported in Figure 6a, with a clear separation between nodes and antinodes induced by the vibrations of the bottom lithium niobate substrate. This solution is here shown to testify that a simple design rule can be that the pressure wave pattern turns out to be more regular when the width of the channel is close to a multiple of the SSAW wavelength. It is important to highlight that the regular distribution of the pressure nodes and antinodes becomes independent of the thickness of the microchannel when its width is equal to a multiple of $\lambda_{SAW}$. In Figure 10, the solutions are reported as relevant to two values of thickness, to be compared with the results previously shown. In this case, the number of pressure nodes moves up to 3, matching the corresponding SSAW nodes in the substrate underneath the channel. This solution provides a slightly lower pressure amplitude of 7 × 10^4^ Pa, as compared to the case reported in Figure 6a. In Figure 10b, the pressure field is plotted for a channel characterized by the same width of 2$\lambda_{SAW}$ and a thickness of 138 *μ*m. As shown in the plot of Figure 8, where the same value of thickness is considered, the pressure field features one horizontal node, as expected. Also in this case, the pressure pattern is mirroring the underneath SSAW, and the pressure magnitude of 5.21 × 10^4^ Pa is near the value for the field shown in Figure 8.

**Figure 11:**
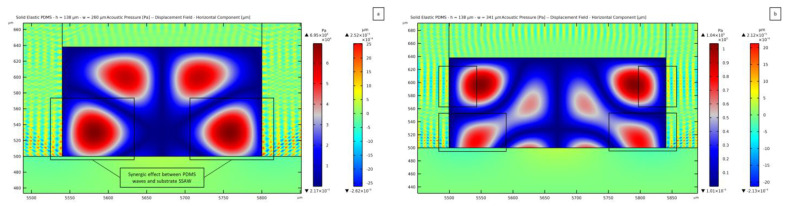
Standing pressure wave field distortion due to the lateral transmission of waves from the PDMS boundaries, relevant to: (**a**) 260 *µ*m wide channel; (**b**) 341 *µ*m wide channel.

Being the pressure distribution independent of the channel width in the case of a thickness of 50 *μ*m, the results here reported refer to a 138 *μ*m thick channel. The parametric analysis has highlighted a distorted pattern of the pressure field within the microchannel in the range of widths between 237 *μ*m and 273 *μ*m, smaller than the value of $\lambda_{SAW}$. Figure 11a shows the pressure field for a 260 *μ*m wide channel. On the lateral walls, the presence of a horizontal wave is highlighted with black boxes at the interface between the fluid and PDMS. The horizontal displacement amplitude in the PDMS has a value of 2.52 × 10^−3^ *μ*m, meaning that the ratio between this horizontal wave amplitude and the one of the SSAW is 5.5.

In the range of widths between 274 *μ*m and 313 *μ*m, the acoustic pressure field within the liquid domain is the same as the one reported for a width of 280 *μ*m in Figure 8.

The pressure field turns out to be distorted for width values between 314 *μ*m and 344 *μ*m, for which two more lateral nodes are expected, $w_{channel}$ being greater than $\lambda_{SAW}$. In this range, the pattern gets distorted with a strange form, similar to what is depicted in Figure 11b for a channel featuring a width of 341 *μ*m. The horizontal motion of the wave in PDMS acts on the fluid domain in a way to cause the emergence of another antinode, more than the two expected from the substrate SSAW transmission, and the same near the corner on top. This synergistic effect leads to interference near the lateral walls, causing the development of the reported pattern. As discussed before, in these cases, the maximum amplitude of the vibration-induced displacements in the PDMS results to be around 5 times greater than the vertical displacement at the substrate surface. This condition, therefore, seems to be a critical one to be attained for the reported secondary waves to overcome the SSAW-induced ones and to lead to detrimental effects on the pressure pattern and the relevant capacity of the device to manipulate the microparticles.

**Figure 12:**
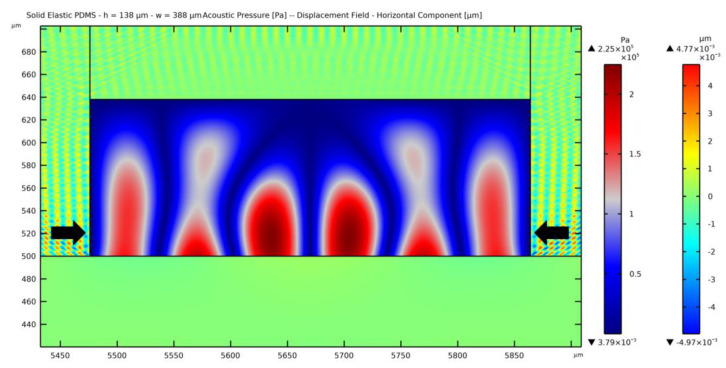
Horizontal component of the displacement field within the PDMS, plotted together with the standing pressure field in case of a 138 *µ*m thick and 388 *µ*m wide fluid domain.

A peculiar solution has been discovered for a width ranging between 345 *μ*m and 420 *μ*m. The interference of the waves causes the presence of five nodes instead of the three expected for the considered width of 388 *μ*m. This pattern seems to be induced by the larger amplitude of the horizontally moving waves in the PDMS with respect to the vertically moving ones from the substrate, as can be seen in Figure 12. The value of the maximum displacement in the vertical direction is 1.09 × 10^−3^ *μ*m, while the one in the horizontal direction is 4.77 × 10^−3^ *μ*m, which is 4.4 times greater and more than 10 times greater compared to the substrate maximum displacement amplitude. The same solution is obtained by solving the problem without accounting for the PDMS wave damping, stating that the distortion can be associated with the geometry of the channel itself.

This second sensitivity analysis associated with the microchannel width, has further shown how the distribution of pressure can be affected by small imperfections in the channel geometry. For example, for a 138 *μ*m thick geometry, a regular distribution is obtained if the width of the channel is designed to be equal to the SSAW wavelength or to a multiple of it. A slight deviation from these values causes the distribution to become distorted, characterized by an unexpected pattern, and so has a critical capability to predict the motion of microparticles. Practically, the expected pressure node and antinode locations correspond to one of the SSAWs; therefore, the expected final position of the particles dispersed inside the fluid will be in the pressure nodes. An unexpected pressure pattern, like the ones shown before, causes the microparticles to move towards pressure nodes in unexpected locations. This feature will be specifically investigated next.

### 3.4. Microparticles Focusing in the Acoustic Pressure Field

To assess the acoustophoretic phenomenon, PS particles have been released in the fluid domain to compute their trajectories induced by the ARF. The reported 2D solutions relevant to the channel cross-section are only intended to show the effect of the acoustic force corresponding to the acoustic radiation.

Results are shown for three channel geometries to assess the capability of particles focusing on the central node. The two pressure patterns reported in Figure 6a, relevant to $h_{channel}$ = 50 *μ*m and $w_{channel}$ = 280 *μ*m, and in Figure 8, relevant to $h_{channel}$ = 138 *μ*m and $w_{channel}$ = 280 *μ*m, have been considered. The distorted pattern reported in Figure 12 has been considered as well, as characterized by $h_{channel}$ = 138 *μ*m and $w_{channel}$ = 388 *μ*m. The study of the motion of the microparticles has been performed with a time-dependent particle tracing analysis. The solutions of the former analyses in the frequency domain have been exploited in terms of standing pressure fields to compute the corresponding values of the ARF. Spherical PS particles with a diameter $d_{p}$ = 8 *μ*m have been released inside the fluid domain. To ease the analyses, the freeze condition has been adopted along the walls of the channel; hence, as soon as the particles touch them, their motion gets frozen. Alternatively, bump conditions on the PDMS walls can be adopted in the analyses. Even if the motion of the particles is modeled for a long enough period, it could happen that all the particles come into contact with the channel walls before the analysis is completed; therefore, the positions of the particles at specific time instants are reported next.

**Figure 13:**
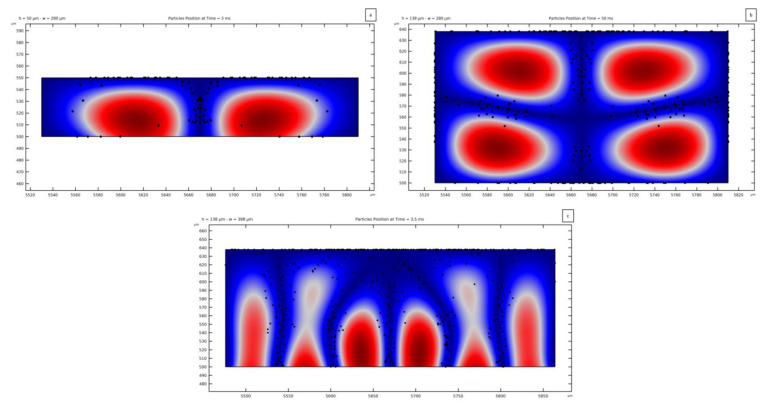
Position of PS microparticles with a diameter of 8 µm, after being displaced by the ARF towards the nodes of the acoustic wave field, in case of PDMS modeled as an elastic solid material. The elapsed time from the release of the particles is (**a**) 3 ms, (**b**) 50 ms and (**c**) 3.5 ms. (**a**) Focusing on a single central pressure node; (**b**) bi-dimensional focusing, exploiting the horizontal pressure node; (**c**) mispositioning caused by unexpected pressure nodes.

Figure 13a shows the focusing of the microparticles in the central pressure node when 80 particles are released from a 5 × 16 grid. As the antinode shape is similar to a lobe, the particles are displaced accordingly, and not all of them move towards the center of the channel, with some sticking on the top wall. The presence of a fluid flowing in the channel causes the hydrodynamic force to focus the microparticles in the central part of the channel cross-section, supporting the acoustic force [7]. In a real case, the particles stopping their motion on the walls can instead bounce back and keep getting focused on the pressure nodes. The time needed to move the particles in the reported positions is computed to be 3 ms, proving how fast the manipulation can be obtained as an equivalent acoustic diffusion time.

Figure 13b highlights the possibility of exploiting this method for three-dimensional manipulation, maybe thanks to multiple actuated structures to transmit pressure waves to the fluid from all directions, as suggested before. In this solution, 304 particles have been released from a 16 × 19 grid. The reported final distribution of particles is obtained in 50 ms and does not undergo relevant modifications afterwards. As can be seen, the presence of other nodes besides the vertical one causes the particles to finally focus on multiple positions.

Mispositioning effects are stressed in the pressure field of Figure 13c, where five pressure nodes are induced by the SSAW and by the secondary PDMS waves transmitted to the fluid. In this case, 464 particles have been released from a 16 × 29 grid. A large set of these particles is shown to be directed towards the top wall and to become frozen there; the remaining particles focus instead on the vertical pressure nodes, as induced by the ARF. The central region of the fluid near the upper wall becomes abounded with microparticles due to a small magnitude of the pressure as compared to the regions near the wall below; this pressure distribution causes the ARF, being proportional to the pressure gradient between separate regions of the pressure field, to show decreasing magnitude if moving upwards in the fluid domain. This result has been reported since it looks fundamental to avoid unexpected pressure patterns in the fluid, with a resulting inability to focus the suspended particles. This outcome accounts for several contributions: The mechanics of the piezoelectric substrate, including the frequency of actuation and the SAW wavelength; the geometry of the channel to generate a regular pressure pattern; and the presence of micro-imperfections causing the distortion of this pattern. The current analyses have testified to the ability to manipulate the microparticles while also exploiting the interference of the fluid with the vibrations in the surrounding media as a premise for a vertical regularity in the pressure field.

### 3.5. Non-Flowing Fluid Modeling of PDMS

Having stated the effect of the secondary waves transmitted by the solid elastic PDMS, a further comparison is here reported to discuss how the solution is affected if the PDMS is modeled as an absorbing, non-flowing fluid.

An impedance boundary condition is adopted at the boundaries of the PDMS domain. The value of the specific impedance applied to the lateral boundaries to mimic air reads $Z_{air}$ = 411.6 Pa∙s/m; the one adopted at the upper boundary to mimic the presence of an additional portion of PDMS reads instead $Z_{PDMS}$ = 1.04 × 10^6^ Pa∙s/m. The acoustic attenuation coefficient that has been associated with the PDMS domain is $\alpha_{PDMS}$ = 7757 dB/m, as obtained by allowing for the experimental data reported in [57]. The acoustic impedance is a specific property of the material, computed as the product of its density by the speed of sound: Z=ρ·c. By comparing the acoustic impedance values at the interface between two different materials, an estimation of the amount of wave energy reflected back or transmitted by this boundary can be obtained. When two materials are characterized by similar values of the acoustic impedance, for instance, water and PDMS, the acoustic wave is more likely to be transmitted through the interface and change the propagation medium. Instead, when the mismatch between the two values is large, as in the case of a PDMS/air interface, a great part of the acoustic waves is reflected back. The percentage of reflected wave energy can be computed using the following equation:

(17) $$R(\%)={\left(\frac{Z_{1}-Z_{2}}{Z_{1}+Z_{2}}\right)}^{2}\cdot 100.$$

The value of R, at the interfaces characterizing the device, provides an indication of the propagation of the acoustic waves. The wave starts propagating on the LiNbO_3_ substrate, featuring an impedance value of $Z_{LN}$ = 1.65 × 10^7^ Pa∙s/m, computed with the Rayleigh wave speed $c_{R}$, and is transmitted to the water inside the microchannel, featuring an impedance of $Z_{i}$ = 1.49 × 10^6^ Pa∙s/m. Substituting $Z_{i}$ and $Z_{LN}$ inside equation 17, the value of R at this interface is 69.6%, which means that the transmitted percentage is T=100%−R = 30.4%. This result can be associated with the leaky-SAW phenomenon due to the lower speed of the acoustic waves in water with respect to the speed of the Rayleigh waves on the substrate. The value of T at the PDMS/water interface is 96.8%, meaning that the acoustic waves can propagate between the fluid and the PDMS almost without reflection.

**Figure 14:**
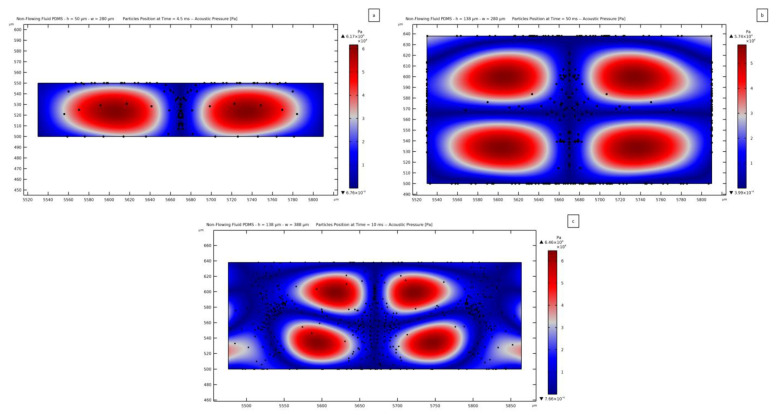
Position of PS microparticles with a diameter of 8 *µ*m, after being displaced by the ARF towards the nodes of the acoustic wave field, in case of PDMS modeled as an absorbing non-flowing fluid. The elapsed time from the release of the particles is (**a**) 4.5 ms, (**b**) 50 ms, and (**c**) 10 ms. These plots are to be compared with those reported in Figure 13.

As a comparison with the solutions reported so far, the same channel geometries considered in Section 3.4 have been considered in the analyses leading to the solutions shown in Figure 14. In this way, both the acoustic pressure field and the effect of the ARF on the positioning of microparticles can be compared. A condition of continuity of the pressure field has been set in the model between the water and PDMS domains, meaning that the Helmholtz equation is solved for both of them with such continuity at the interface. This condition has not been imposed for the solid elastic PDMS model, as the elastic response of the PDMS to the SSAW stimulus has been computed, and then the elastic waves have been transmitted to the fluid in the microchannel.

For the channel 50 *μ*m thick and 280 *μ*m wide, the acoustic pressure distribution results are similar to the already reported solution, with one central pressure node and two lateral antinodes, to resemble the shape of the SSAW in the substrate. The amplitude of the acoustic pressure within the fluid domain is 6.17 × 10^4^ Pa, amounting to 73% of the value obtained with the solid elastic PDMS case. In Figure 14a, the position of the microparticles is reported; it can be seen that the same distribution of particles reported in Figure 13a has been obtained. The ARF pushes the particles mainly towards the central pressure node, with only a few particles displaced towards the upper and lateral surfaces.

Figure 14b reports the acoustic pressure in the case of a channel 138 *μ*m thick and 280 *μ*m wide. Also in this case, the acoustic pressure distribution is similar to the already discussed one, with one vertical node, one horizontal node, and four antinodes. By comparing this solution with the one reported in Figure 13b, it can be seen that the horizontal node seems to be flatter in the present solution of Figure 14b. Anyway, the motion of the microparticles turns out to be the same as the one reported in Figure 13b.

The solution for the case featuring a channel 138 *μ*m thick and 388 *μ*m wide is instead much different from that of the other model. The acoustic pressure distribution shown in Figure 14c is similar to the one provided by the waves in the substrate; therefore, the central pressure node sets in together with a horizontal wavy node, no more flat than in the solution reported in Figure 14b. Four antinodes also show up, as expected for this channel thickness. The shape of the pressure wave in the central portion of the channel is similar to the one reported in Figure 13b and Figure 14b. Near the lateral walls, a less regular solution has been found. This effect can be related to the width of the channel being larger than $\lambda_{SAW}$ and smaller than 2$\lambda_{SAW}$; therefore, the successive antinode cannot be fully enforced by the substrate. Similarly to what was observed in Section 3.3, the best way to generate a regular acoustic pressure distribution seems to be provided by designing a microchannel whose width is a multiple of $\lambda_{SAW}$, so that the SSAW can be properly transmitted. This rule is not meant to be generally valid; it is just reported as a consideration for the results obtained with the reported model. The contribution of the waves propagating within the PDMS, if modeled as a solid elastic material, can be clearly distinguished by comparing Figure 13b and Figure 14b. The waves in the PDMS structure, being greater in magnitude than the ones coming from the substrate, provide an additional antinode to the pressure field and cause two more nodes if compared to the field in Figure 14b. An additional effect caused by the waves in the PDMS is related to the amplitude of the pressure wave within the channel, which results in a greater magnitude than the one achieved for a non-flowing fluid model. Specifically, it turns out to be equal to 6.46 × 10^4^ Pa in the latter analysis, only 29% of the value obtained with the solid elastic PDMS solution. The two solutions reported in Figure 13c and Figure 14c have led to two different particle-focusing positions. Considering the SSAW, the expected pressure distribution is more similar to the one obtained by modeling the PDMS as an absorbing, non-flowing fluid. This model, the other way around, does not take into account the propagation of elastic waves within the PDMS, which causes the generation of a more distorted pressure field. Therefore, the two results highlight the importance of modeling the solid nature of PDMS to fully assess the behavior of the material in response to actuation of the substrate surface.

## 4. Conclusions

The present study has highlighted the mechanism of triggering a standing acoustic pressure wave generated with an SSAW, propagating in a piezoelectric substrate, and interacting with waves forming inside a compliant polymeric microchannel.

The generation, propagation, and interference of the TSAWs have been modeled within a time-dependent analysis. The leaky wave phenomenon and the transmission of a standing pressure wave have been assessed by allowing for the coupling between mechanics and acoustics. A sensitivity analysis has been conducted to investigate how the standing pressure field can be modified by the geometry of the fluid domain, characterized by the width and thickness of its rectangular cross-section. The result to be achieved, is represented by a structured pressure distribution made of nodes and antinodes, as a correct/accurate transmission to the fluid of the SSAW pattern. By modeling the PDMS as an elastic material, secondary waves in the microchannel structure have been shown to be generated and to affect the pressure waves in the fluid. These waves excite the fluid from all the solid/fluid interfaces, causing the modification of the pressure nodes and antinodes distribution when the amplitude of PDMS vibration-induced waves overcomes a certain threshold relative to the SSAW amplitude.

The trajectories of particles released into the fluid have been simulated. The ARF, which arises from a pressure difference between different zones of the standing acoustic wave, acts on the particles and moves them from their original positions towards the nodes. Different final positions have been reported at varying sizes of the microchannel. This outcome can be used as a basis for the design of SAW-based devices to lead to accurate acoustic manipulation of microparticles or even cells.

Finally, a comparison of the acoustic pressure field within the microchannel section has been reported by modeling the PDMS as an absorbing, non-flowing fluid to account for the soft nature of the material. The differences between the two model results have been highlighted by showing how microparticles can be displaced differently. The effect of the secondary waves generated within the PDMS has therefore been assessed.

The integration of multiple wave sources on a single device could be addressed as a solution to compensate for the vibrations in the PDMS microchannel and reduce the generation of unexpected pressure patterns. Moreover, an acoustofluidic system in which the acoustic waves are induced from more than one direction, such as the case here presented, can be designed to lead to an engineered pressure distribution, featuring enhanced capability in 3D particle manipulation.

## Figures and Tables

**Table 1:** Adopted material properties.

Material	Property	Value	Unit
Y-cut lithium niobate (LiNbO_3_)	Density $\rho_{s}$	4700	kg/m^3^
	Elasticity coeff. $c_{11}$	203	GPa
	Elasticity coeff. $c_{33}$	243	GPa
	Elasticity coeff. $c_{55}$	59.9	GPa
	Elasticity coeff. $c_{13}$	74.9	GPa
	Coupling coeff. $e_{31}$	0.19	C/m^2^
	Coupling coeff. $e_{33}$	1.31	C/m^2^
	Coupling coeff. $e_{15}$	3.69	C/m^2^
	Permittivity coeff. $\varepsilon_{11}$	43.6	-
	Permittivity coeff. $\varepsilon_{33}$	29.2	-
	Rayleigh wave speed $c_{R}$	3505	m/s
PDMS	Density $\rho_{PDMS}$	970	kg/m^3^
	Young’s modulus $E_{PDMS}$	750	kPa
	Poisson’s ratio $\nu_{PDMS}$	0.49	-
	Isotropic damping factor [54] $\eta_{s}$	0.001	-
	Speed of sound $c_{PDMS}$	1076.5	m/s
	Specific acoustic impedance $Z_{PDMS}$	1.04 × 10^6^	Pa∙s/m
	Acoustic attenuation coeff. $\alpha_{PDMS}$	7757	dB/m
Air	Specific acoustic impedance $Z_{air}$	411.6	Pa∙s/m
	Speed of sound $c_{air}$	343	m/s
Water	Acoustic attenuation coeff. $\alpha_{i}$	36.67	dB/m
	Density $\rho_{i}$	998	kg/m^3^
	Speed of sound $c_{i}$	1500	m/s
	Compressibility $\kappa_{i}$	448	TPa^−1^
	Specific acoustic impedance $Z_{i}$	1.49 × 10^6^	Pa∙s/m
Polystyrene (PS)	Density $\rho_{p}$	1050	kg/m^3^
	Compressibility $\kappa_{p}$	249	TPa^−1^

## Data Availability

Not applicable.
